# The hidden Markov chain modelling of the COVID-19 spreading using Moroccan dataset

**DOI:** 10.1016/j.dib.2020.106067

**Published:** 2020-07-24

**Authors:** Abdelghafour Marfak, Doha Achak, Asmaa Azizi, Chakib Nejjari, Khalid Aboudi, Elmadani Saad, Abderraouf Hilali, Ibtissam Youlyouz-Marfak

**Affiliations:** a Laboratory of Health Sciences and Technology, Higher Institute of Health Sciences, Hassan First University of Settat, Morocco; b Higher Institute of Nursing Professions and Health Technology of Rabat, Morocco; c Epidemiology, Clinical Research and Community Health, Faculty of Medicine and Pharmacy of Fez, University Sidi Mohammed Ben Abdellah, Fez, Morocco; d International School of Public Health, Mohammed VI University of Health Sciences (UM6SS), Casablanca, Morocco

**Keywords:** COVID-19 spreading, Hidden Markov chain, Statistical modelling, forecast

## Abstract

The World Health Organization (WHO) declared in March 12, 2020 the COVID-19 disease as pandemic. In Morocco, the first local transmission case was detected in March 13. The number of confirmed cases has gradually increased to reach 15,194 on July 10, 2020. To predict the COVID-19 evolution, statistical and mathematical models such as generalized logistic growth model [1], exponential model [2], segmented Poisson model [3], Susceptible-Infected-Recovered derivative models [4] and ARIMA [5] have been proposed and used. Herein, we proposed the use of the Hidden Markov Chain, which is a statistical system modelling transitions from one state (confirmed cases, recovered, active or death) to another according to a transition probability matrix to forecast the evolution of COVID-19 in Morocco from March 14, to October 5, 2020. In our knowledge the Hidden Markov Chain was not yet applied to the COVID-19 spreading. Forecasts for the cumulative number of confirmed, recovered, active and death cases can help the Moroccan authorities to set up adequate protocols for managing the post-confinement due to COVID-19. We provided both the recorded and forecasted data matrices of the cumulative number of the confirmed, recovered and active cases through the range of the studied dates.

**Specifications table**

**Table:** 

**Subject**	Epidemiology
**Specific subject area**	Statistical model applied to the COVID-19 pandemic data to forecast the cumulative number of the confirmed, recovered, active and death cases
**Type of data**	Table Graph
**How data were acquired**	The data were acquired from the official website (https://covid19-geomatic.hub.arcgis.com/) Instruments: The R package “markovchain” was used
**Data format**	Data are in raw format and provided in an Excel file
**Parameters for data collection**	The data matrix consists of the reported cumulative number of the COVID-19 confirmed, recovered, active and death cases
**Description of data collection**	Data were obtained daily at 6 p.m. from the official report of health ministry for the pandemic situation. All data were collected between March 13 and July 10, 2020 yielding to a matrix data of 120 × 4 observations.
**Data source location**	Institution: Laboratory of Health Sciences and Technology, Higher Institute of Health Sciences, Hassan First University of Settat City: Settat Country: Morocco
**Data accessibility**	Reported and forecasted data were provided with the article in supplementary Excel file

## Value of the data

• These data provide forecasts for the cumulative number of confirmed, recovered, active and death cases, which is important for both monitoring and control the COVID-19 spreading.
• Basing on the predicted values up to October 5, 2020, the authorities can benefit from these data to set up adequate protocols for the post-confinement.
• These data might be used by other researchers for comparison and further meta-analysis of the COVID-19 worldwide spreading.
• These data may complete the statistical and mathematical models developed until now for modelling the COVID-19 evolution, which allow more understanding, modelling and managing epidemic crisis.

## 1 Data description

**Fig. 1:**
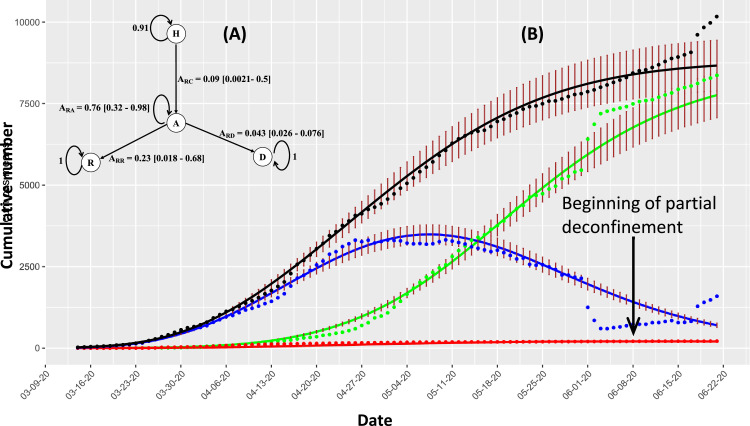
(A) The Markov chain diagram for Covid-19 forecasting. A_RC_: Average rate of confirmed cases, A_RD_: Average rate of death cases, A_RR_: Average rate of recovered cases and A_RA_: Average rate of active cases; H: Healthy, A: Active, R: Recovered and D: Death. (B) The observed (dot) and predicted (solid line) cumulative number from March 14, to Jun 22, 2020. Cumulative confirmed cases (black), cumulative recovered cases (green), cumulative active cases (blue) and cumulative number of deaths (red).

**Fig. 2:**
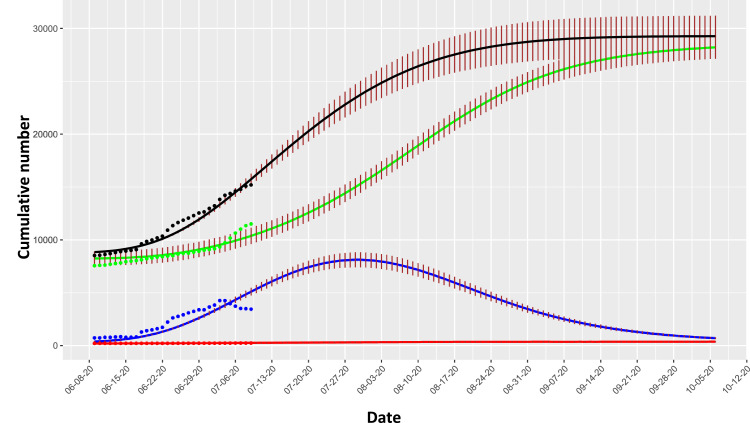
The observed (dot) and forecasts (solid line) cumulative number from Jun 8, to October 5, 2020. Cumulative confirmed cases (black), cumulative recovered cases (green), cumulative active cases (blue) and cumulative number of deaths (red).

Fig. 1, Fig. 2 show the predictions up to October 5, 2020. The daily forecasts for the cumulative number of confirmed, recovered, active cases and the cumulative number of deaths were compared to that reported by the ministry of health [6, 7] date to date as new data become available. The cumulative number of cases reported in Morocco on July 10, 2020 (end of the data collection) was 15,328. The predicted number of confirmed cases at this date was 15,731 [15,257; 16,204]. Both the observed and predicted number of confirmed cumulative cases increased following a logistic distribution. Regarding the number of cumulative deaths, in July 10, the observed number was 243 and the predicted number was 255 [252; 258]. The cumulative active cases increased to reach a maximum number on May 4, and decreased progressively with slight fluctuations around the predicted curve (Fig. 1A). In Jun 9, the minimum observed value for active cumulative number was 733 (the predicted number was 1419 [1288; 2162]). From June 12, Fig. 1A shows an increase for the number of active cases due to an increase in the confirmed cases. In Morocco, a partial deconfinement authorizing displacement between some cities began from June 10. Taking into account the post-confinement, Fig. 2 depicted forecasts from Jun 8, to October 5, 2020. The supplementary file is an Excel tabulated data containing the predicted cumulative number of confirmed, recovered, active and death cases and their 95% confidence intervals. Also, the supplementary file provides the reported cumulative number from the Ministry of health of Morocco.

## 2 Experimental design, materials and methods

Updates number of screening test for COVID-19 and the cumulative number of reported confirmed, recovered and death cases were obtained daily at 6 p.m. from the official report of health ministry for the pandemic situation [6, 7]. All data were collected between March 13 and July 10, 2020 yielding to a matrix data of 120 × 4 observations. In order to forecast the cumulative number of confirmed, recovered, active and death cases, our modelling of the COVID-19 spreading in Morocco started at March 13, 2020 using the following steps:

1) For any given day_(j)_ we calculated:
(i) The rate of confirmed cases (RC) as the number of new cases divided by the cumulative number of cases in day_(j)_.
(ii) The recovered rate (RR) as the cumulative number of recovered patients divided by the cumulative number of cases in day_(j)_.
(iii) The death rate (RD) as the cumulative number of deaths divided by the cumulative number of cases in day_(j)_.
(iv) The rate of active cases (RA) (patients under treatment but not completely recovered) as the difference between the cumulative number of confirmed cases and recovered patients divided by the cumulative number of cases in day_(j)_.
2) The averages of the RC (A_RC_), RR (A_RR_), RD (A_RD_) and RA (A_RA_) rates were calculated from the 120 × 4 observations data matrix.
3) We considered a Markov process with the state space (healthy "H", active "A", recovered "R" and death "D"). The probabilities of transitioning were the averages’ rates (A_RC_, A_RR_, A_RD_ and A_RA_) (Fig. 1A).
4) To forecast the cumulative number of confirmed, recovered, active and death cases, we used dynamic modelling. The “today_(j)_” state of the (“H”, “A”, “R”, “D”) system is multiplied iteratively by the transition matrix to predict the number of cases for “day_(_*_j_* _+_ _1)_”. At each iteration, the estimated values for “day_(j)_” were used to forecast the number of cases on “day_(_*_j_* _+_ _1)_” and so on.

We started the process by fixing the initial condition to the first data point (March 13, 2020). We had 8 cumulative confirmed cases, 1 recovered and 1 death. The forecasts were estimated for March 14 to October 5, 2020. The RA and RR rates were assumed to obey a logistic distribution. While the RC and RD rates were simulated from exponential and normal distributions, respectively. We conducted 10,000 simulations and the 95% confidence intervals were estimated for each forecast of daily cumulative number of confirmed, recovered, active and death cases.

## Declaration of Competing Interest

The authors declare that they have no known competing financial interests or personal relationships which have, or could be perceived to have, influenced the work reported in this article.

## Appendix Supplementary materials

Image, application 1

Image, application 2
